# Human bone marrow-derived and umbilical cord-derived mesenchymal stem cells for alleviating neuropathic pain in a spinal cord injury model

**DOI:** 10.1186/s13287-016-0295-2

**Published:** 2016-03-08

**Authors:** Mahmoud Yousefifard, Farinaz Nasirinezhad, Homa Shardi Manaheji, Atousa Janzadeh, Mostafa Hosseini, Mansoor Keshavarz

**Affiliations:** Electrophysiology Research Center, Tehran University of Medical Sciences, Tehran, Iran; Department of Physiology, School of Medicine, Tehran University of Medical Sciences, Tehran, Iran; Physiology Research Center, Iran University of Medical Sciences, Tehran, Iran; Department of Physiology, Iran University of Medical Sciences, Tehran, Iran; Department of Physiology, Shahid Beheshti University of Medical Sciences, Tehran, Iran; Neuroscience Research Center, Shahid Beheshti University of Medical Sciences, Tehran, Iran; Department of Epidemiology and Biostatistics, School of Public Health, Tehran University of Medical Sciences, Tehran, Iran; Pediatric Chronic Kidney Disease Research Center, Childrens Hospital Medical Center, Tehran University of Medical Sciences, Tehran, Iran

**Keywords:** Spinal cord injuries, Neuropathic pain, Stem cells, Electrophysiologic techniques, Wind up

## Abstract

**Background:**

Stem cell therapy can be used for alleviating the neuropathic pain induced by spinal cord injuries (SCIs). However, survival and differentiation of stem cells following their transplantation vary depending on the host and intrinsic factors of the cell. Therefore, the present study aimed to determine the effect of stem cells derived from bone marrow (BM-MSC) and umbilical cord (UC-MSC) on neuropathic pain relief.

**Methods:**

A compression model was used to induce SCI in a rat model. A week after SCI, about 1 million cells were transplanted into the spinal cord. Behavioral tests, including motor function recovery, mechanical allodynia, cold allodynia, mechanical hyperalgesia, and thermal hyperalgesia, were carried out every week for 8 weeks after SCI induction. A single unit recording and histological evaluation were then performed.

**Results:**

We show that BM-MSC and UC-MSC transplantations led to improving functional recovery, allodynia, and hyperalgesia. No difference was seen between the two cell groups regarding motor recovery and alleviating the allodynia and hyperalgesia. These cells survived in the tissue at least 8 weeks and prevented cavity formation due to SCI. However, survival rate of UC-MSC was significantly higher than BM-MSC. Electrophysiological evaluations showed that transplantation of UC-MSC brings about better results than BM-MSCs in wind up of wide dynamic range neurons.

**Conclusions:**

The results of the present study show that BM-MSC and UC-MSC transplantations alleviated the symptoms of neuropathic pain and resulted in subsequent motor recovery after SCI. However, survival rate and electrophysiological findings of UC-MSC were significantly better than BM-MSC.

## Background

According to the International Association for the Study of Pain (ISAP), neuropathic pain is a pain caused by damage or diseases affecting the central or peripheral nervous system. Following spinal cord injury (SCI) most patients suffer from long-lasting moderate to severe pain [1–4]. Existing treatments for reducing neuropathic pain have low efficiency in the majority of patients. These treatments include surgical decompression, drug therapy, and palliative care; even new drugs only reduce the pain by 50 % in a quarter of the patients [5]. These therapeutic strategies are purely conservative, and side effects caused by long-term use of the drugs are a great obstacle for applying this method of pain reduction [6, 7]. Neuropathic pain will persist unless the damaged area is healed or pain reduction pathways are amplified. Therefore, researchers are looking to repair the damaged nerve cells.

Intrinsic regeneration of the damaged nerves in the central nervous system is limited, so scientists are trying to build new nervous contacts at the site of injury to reduce the neuropathic pain [8]. Thus, cell transplantation is thought to be a suitable treatment for SCI. As a result, in recent years ample research has been done in this field, the result of which shows powerful influence of stem cell transplantation in functional recovery after SCI [9, 10]. These studies showed that stem cells are able to proliferate and differentiate into nerve cells such as mature neurons or glial cells under special circumstances [11]. However, survival and differentiation of stem cells following their transplantation varies depending on the host and intrinsic factors of the cell [12, 13]. Based on these findings, it can be stated that the fate of transplanted cells in vivo varies with the intrinsic characteristics of the cells and site of transplantation [14]. However, the optimal source of stem cells is a controversial issue for treating SCI [15, 16].

Mesenchymal stem cells (MSCs) are the main source of cell therapy because of their capability of differentiating into multiple cell types, including blood, adipose tissue, connective tissues, and so forth [17–19]. These cells can easily grow in vitro and exhibit intriguing immunomodulatory properties, non-teratogenicity, and multi-potentiality with high genetic stability. MSCs can maintain regenerative capacity after cryopreservation, improve synaptic transmission, and promote neuronal networks [20–24]. These properties make MSCs prime candidates for various therapeutic applications especially for nervous system repair.

Different sources can be used to isolate MSCs. Some of these resources include umbilical cord [25], placenta [26], bone marrow [27] and adipose tissue [28]. Bone marrow and umbilical cord are rich sources of MSCs. Transplantation of bone marrow-derived mesenchymal stem cells (BM-MSCs) to the injured spinal cord resulted in a significant improvement in sensorimotor of the hindlimb and reduced cavity formation, and show substantial immunosuppressive, anti-proliferative, anti-inflammatory, and anti-apoptotic properties [29]. Human umbilical cord-derived mesenchymal stem cells (UC-MSCs) can differentiate into various neural cells and have beneficial effects on improving functional recovery after SCI. Compared with BM-MSCs, UC-MSCs have higher expansion ability, robust proliferation capacity, and lower risk of bacterial/viral infection [30], while some reports have shown that UC-MSCs evoked an immune response when injected into injured tissues [31].

The difference in the properties of the BM-MSCs and UC-MSCs may have impact on their efficacy in improving SCIs. However, the effectiveness of these cells in reducing neuropathic pain is not fully understood. Therefore, the present study aimed to determine the effect of stem cells derived from bone marrow and umbilical cord on SCI-induced neuropathic pain, and to identify the stem cell population with the highest survival and effectiveness in transplantation to the site of nerve injury. We selected the rat model because multiple studies of stem cell therapy have been performed in the rat injured spinal cord, and ethical issues do not yet allow us to transplant these cells into human spinal cord.

## Materials and methods

### Study design

The present experimental study aims to compare the effect of transplanting UCMSCs and BM-MSCs on functional recovery and neuropathic pain caused by SCI. The protocol of the present study was approved by the Tehran University of Medical Sciences Ethics Committee. The researchers adhered to the principles of the Helsinki Declaration and the principles of using laboratory animals as suggested in the National Institutes of Health Guide for Care and Use of Laboratory Animals (Publication No. 85–23, revised 1985) over the course of the study. Most of the materials were obtained from Sigma-Aldrich Company, Germany. For all other cases, the relevant company is given.

### Studied animals

Male Wistar rats (n = 72) with a weight range of 140–160 g were used and randomly divided into six groups (12 animals in each group) (Table 1). The animals were obtained from the Laboratory Animal Breeding Center of Iran University of Medical Sciences. All animals were kept in special cages for at least 2 weeks before the initiation of the study for adaptation to the environment. They had free access to water and food (temperature 21 ± 1 °C; 12-hour light/dark cycle). All behavioral tests were performed between 10:00 am and 2:00 pm at room temperature.

**Table 1:** The study groups

Experimental group		Treatment protocol
Control		Healthy animals without treatment
Sham		Laminectomy without SCI induction
SCI		Laminectomy + SCI induction
Vehicle		Laminectomy + SCI + intraspinal injection of cell culture media
BM-MSC		Laminectomy + SCI + intraspinal injection of BM-MSCs
UC-MSC		Laminectomy + SCI + intraspinal injection of UC-MSCs

*BM-MSC* bone marrow-derived mesenchymal stem cell, *SCI* spinal cord injury, *UC-MSC* umbilical cord-derived mesenchymal stem cell

### Cell culture

The cells were of human source in this study. All samples were obtained with written, informed consent in accordance with the Tehran University of Medical Sciences ethics committee requirements. BM-MSCs were bought from the Royan Institute. The cells were kept in an incubator at 37 °C, 90 % humidity, and 5 % CO_2_. They were cultured in cell culture flasks containing DMEM/F_12_ (Dulbecco's modified Eagle's medium/F12; Gibco, Australia), fetal bovine serum 10 % (Gibco) and a combination of penicillin (100 IU/ml), streptomycin sulfate (160 μg/ml), and amphotericin B (10 μg/ml). The medium was changed every 3 days.

UC-MSCs were isolated from Wharton's jelly as follows. After obtaining the mother’s consent, the umbilical cord of a healthy infant born by C-section (n = 2) was brought to the cell culture laboratory under sterile conditions and in HBSS (Hank's Balanced Salt Solution) containing penicillin (100 IU/ml), streptomycin sulfate (160 μg/ml), and amphotericin B (10 μg/ml). UC-MSCs were isolated under sterile conditions. After washing the umbilical cord with 70 % alcohol and phosphate-buffered saline (PBS), amnion and umbilical cord blood vessels were removed accurately and the remaining matrix was chopped into pieces, about 5 mm in diameter. The pieces were moved to 35 × 10 mm petri dishes, and 1 ml DMEM/F_12_ with 20 % fetal bovine serum (Gibco), penicillin (100 IU/ml), and streptomycin sulfate (150 μg/ml) were added. After 10–15 days of culture and keeping cells in an incubator, cell buds were identified next to the pieces. After seeing cell buds, Wharton’s gel pieces were removed from the medium and cell culture continued until the cells reached more than 80 % confluence.

Before transplantation, the surface antigens of the cells were checked using a flow cytometry technique to be sure of their stem cell status. Mesenchymal cells should be negative for CD45 and CD14 but should express CD105, CD29, CD90, and CD44 [32, 33].

### SCI induction

A clip compression model was used to induce SCI. This method was introduced in 1978 [34] and validated in subsequent studies [35–37]. Briefly, rats weighting 140–160 g were anesthetized using ketamine (80 mg/kg) and Xylazin (10 mg/kg). After shaving the hair on their back, a 2-cm long incision was made in the T6–T8 area. Muscles were set aside and the spinal cord was exposed with laminectomy. Afterwards, and with much caution, the spinal cord was compressed using a calibrated aneurysm clip providing 20 g/cm^2^ pressure. The force of the clip was measured as described previously [38]. The clip was removed after 60 seconds and muscles and skin were sutured separately to close the operation site. Since the animals were incapable of emptying their bladder voluntarily after injury induction, their bladder was emptied at least twice a day until they were able to do so themselves.

### Stem cell transplantation

A week after SCI induction, the animals were prepared for transplantation. They were anesthetized using ketamine (80 mg/kg) and Xylazin (10 mg/kg) and their spinal cord was exposed at the T6–T8 area in the same way as stated above in the SCI induction section. About 1 million cells in a 10-μl volume were then transplanted into the dorsal horn of the spinal cord in two injections, 0.5 mm rostral and caudal of the lesion at a depth of 1 mm below the dorsal surface at a rate of 1 μl/min, using a glass micropipette attached to a stereotaxic injector. Subsequently, the muscles and skin were sutured and the animals were returned to their cages. To confirm the number of cells, the sample was prepared and cell count was performed using trypan blue staining.

### Behavioral evaluations

Behavioral tests were carried out every week for 8 weeks after SCI induction. The Basso, Beattie, and Bresnahan (BBB) locomotor scoring scale [39] was used to rate the hind limb motor function. The rats were placed in a container 120 cm in diameter and were studied and rated for 4 minutes. The locomotor behavior of the animals, including hind-limb motor function, weight-bearing, limb coordination, and walking, was assessed and scored.

To evaluate sensory function, four behavioral tests were used, the details of which have been described in a previous study by the authors [40]. In summary, mechanical allodynia was evaluated using the von-Frey test. Eight von-Frey filaments of different diameters were used in an up and down manner to assess the withdrawal threshold of the animal; 50 % withdrawal threshold was then calculated based on the responses. Cold allodynia was evaluated using the acetone test. In this test, about 100 μl acetone was pushed onto the hind paw of the animal. The test was repeated five times for each paw with 1-minute time intervals, and the number of withdrawals was considered as the response and presented as a percentage of the total. Mechanical hyperalgesia was evaluated (Randall-Selitto test) using an analgesia meter. In this test, increasing mechanical tension was applied to both hind limbs with at least 1-minute intervals and their average was recorded. Heat hyperalgesia was assessed by thermal stimulation of the animal’s hind paw (Plantar test). The test was repeated three times and the average time was considered as the animal’s withdrawal latency. A 25-second cut-off was used for stopping stimulation to avoid tissue damage.

### Electrophysiological evaluation

At the end of week 8, a single-unit recording of the dorsal horn of the spinal cord was obtained to evaluate the electrical function of neurons. For this purpose, the animals were deeply sedated (60 mg/kg pentobarbital) and their body temperature was kept at 37 °C over the course of the recording. L1–L2 lamina was then removed for electrophysiological recording. The recording site deviated 0.6 mm from the middle (to the side of the spinal cord) and its depth was 300–700 μm. This depth was selected due to the presence of wide dynamic range (WDR) neurons. These cells receive input from all three types of sensory fibers (Aβ, Aδ, and C), and therefore respond to the full range of stimulation, from light touch to noxious pinch, heat, and chemicals [41]. For each animal, one neuron was evaluated. Electrical stimulation was used to induce responses. Neural response recording started when a stable response from the neuron lasted for at least 1 minute. The spikes of action potential were recorded and their frequency was counted in four delayed window periods after stimulation: 0–20 ms (for Aβ fibers), 20–90 ms (for Aδ fibers), 90–300 ms (for C fibers), and 300–800 ms (for post-discharge). Finally, wind-up phenomenon was calculated based on Jergova et al. [42].

### Histological evaluation

The spinal cord was prepared for tissue evaluation in week 8. After transcardial perfusion, the spinal cord was fixated in 4 % paraformaldehyde in 0.1 molar phosphate buffer, pH 7.4, for 24 hours. Then it was kept in 10 %, 20 % and 30 % sucrose solutions for 24 hours each and prepared for serial cross-sectionalizing. Luxol fast blue (LFB) staining was performed to determine the volume of the injury; 20-μm diameter sections were stained [43]. The sections were then observed under a light microscope. The size of the cavity was divided by the total size of the spinal cord section (three sections that had the biggest cavity for each animal; three animals in each group) and were presented as percentages. The data were analyzed with ImageJ software (Wayne Rasband, National Institutes of Health, USA).

Eight weeks after cell transplantation, survival of the cells was evaluated by the aid of immunohistochemistry. The cryostat sections (20 μm) were permeabilized in PBS-T (PBS containing 0.1 % Triton X-100) for 10 minutes and blocked with 10 % fetal bovine serum in PBS-T for 1 hour, and then incubated overnight at 4 °C with the primary antibodies against mouse monoclonal antibody against human nuclei (HuNu; Chemicon Inc., Pittsburgh, PA, USA). Then lamels were washed and goat anti-rabbit IgG conjugated with Alexa-Fluor 594 (Molecular Probes, Eugene, OR, USA) secondary antibodies were added in a 1:100 dilution and incubated at 37 °C for 1 hour. The nucleus of the host cell was also stained using DAPI (4',6-diamidino-2-phenylindole; Molecular Probes, Eugene, OR, USA) and the results were assessed using an Olympus DP72 florescent microscope. In this type of staining, transplanted cells are stained green. All sections were stained (three animals in each groups). The survival rates were calculated based on following formula:

$$ Survival\kern0.5em  rate\kern0.5em \left(\%\right)\kern0.5em =\kern0.5em \frac{total\kern0.5em  number\kern0.5em  of\kern0.5em  survived\kern0.5em  cells\kern0.5em  in\kern0.5em 8th\kern0.5em  week}{total\kern0.5em  number\kern0.5em  of\kern0.5em  transplanted\kern0.5em  cells}\kern0.5em \times \kern0.5em 100 $$

### Statistical analyses

Data were analyzed by SPSS version 21.0 and are presented as mean and standard error. To compare the data gathered from behavioral evaluations of the different groups, two-way analysis of variance with Bonferroni post-hoc test was used, and for assessment of electrophysiological assessment and histological assays, one-way analysis of variance was used. In all analyses, *p* < 0.05 was considered as significant.

## Results

### Mesenchymal cell characteristics

**Fig. 1:**
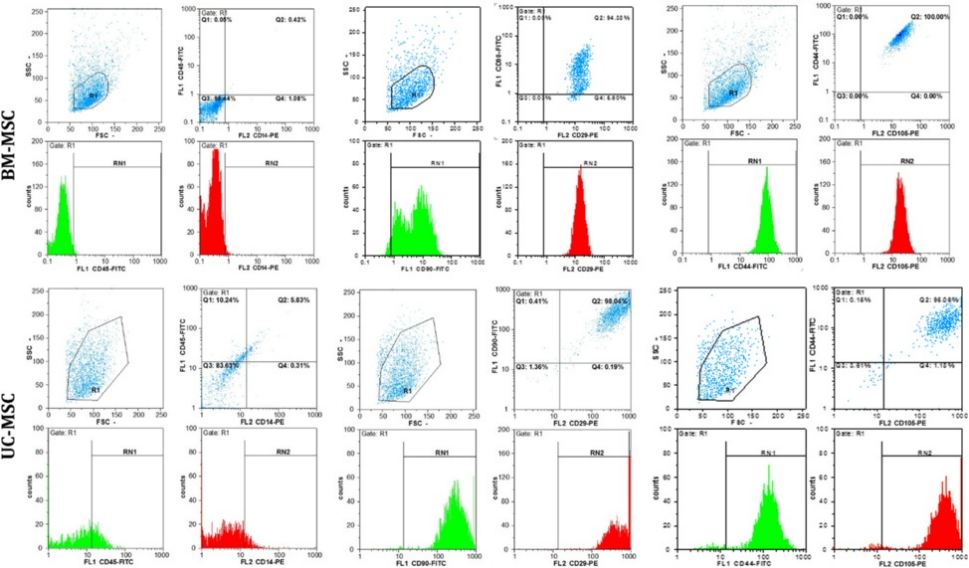
Immunophenotype results of cells derived from human bone marrow mesenchymal stem cells (*BM-MSCs*) and umbilical cord mesenchymal stem cells (*UC-MSCs*). All cells were positive for CD29, CD44, CD90, and CD105, but negative for CD14, and CD45

After isolation, BM-MSCs and UC-MSCs adhered to the bottom of the flask and formed colonies. They became spindle-shaped and suspended cells were removed by medium change. Figure 1 shows the surface antigen profile of these spindle-shaped cells evaluated using flow cytometry. All cells were negative for CD45 and CD14. BM-MSCs were positive for CD44 and CD105 and UC-MSCs expressed CD29 and CD90.

### Behavioral evaluations

#### Motor recovery

**Fig. 2:**
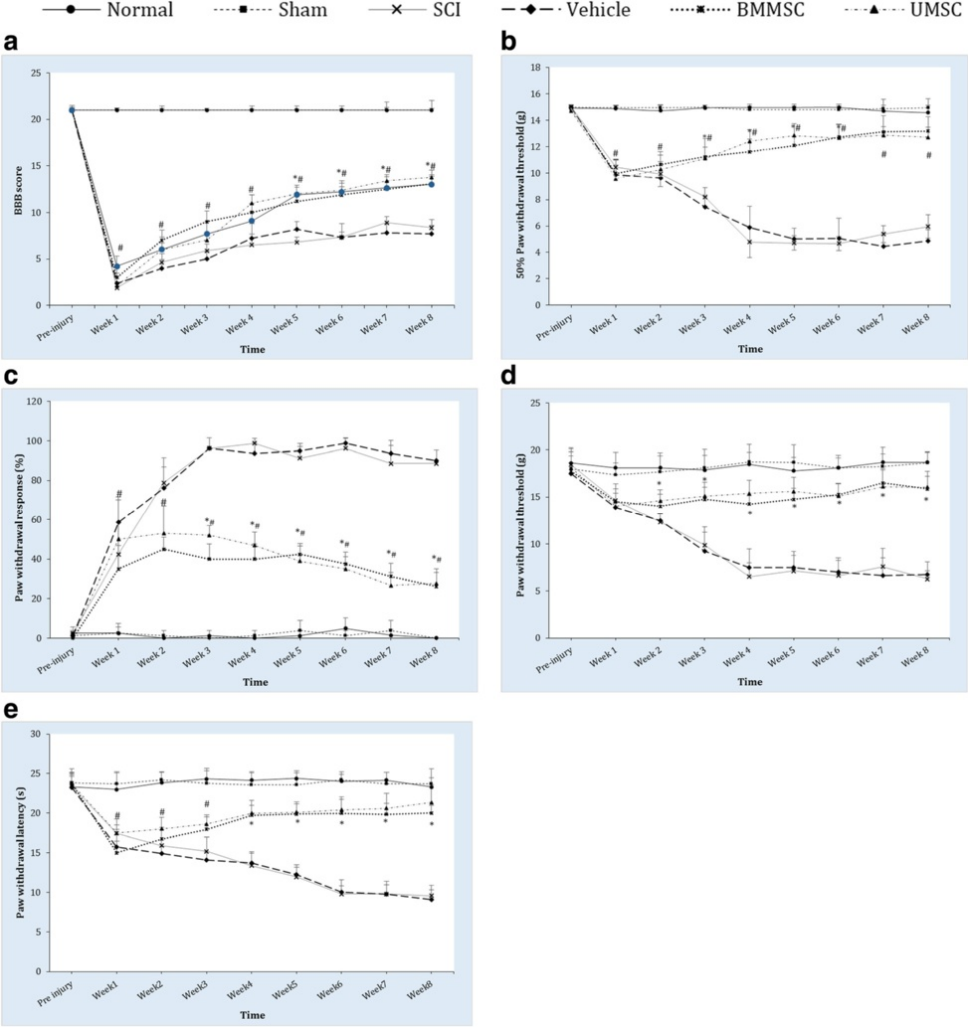
Effect of intraspinal transplantation of human bone marrow mesenchymal stem cells (*BMMSCs*) and umbilical cord mesenchymal stem cells (*UMSCs*) on motor function recovery (**a**), mechanical allodynia (**b**), cold allodynia (**c**), mechanical hyperalgesia (**d**), and heat hyperalgesia (**e**). Data are expressed as means ± SEM (n = 12 in each group). The paw withdrawal thresholds are significantly increased following stem cell transplantation compared to spinal cord injured animals (*SCI*). *p < 0.01, versus SCI group; ^#^ *p* < 0.01, versus sham animals. *BBB* Basso, Beattie, and Bresnahan

After SCI induction, the locomotor score of the animals significantly decreased compared to the sham group (df: 8, 63; F = 79.6; *p* < 0.001). Stem cell transplants did not lead to significant improvement until the fourth week after SCI. However, from weeks 5 to 8 of the study, BM-MSC and UC-MSC transplantations led to progressive improvement of motor recovery in animals compared to the vehicle group (df: 8, 63; F = 366.4; *p* < 0.0001). No difference was seen between the two cell groups regarding motor recovery (*p* > 0.99) (Fig. 2a).

#### Mechanical allodynia

SCI resulted in a significant decrease in 50 % paw withdrawal threshold of the animals (df: 8, 63; F = 48.6; *p* < 0.001). This decline continued in the vehicle and SCI groups until week 4 and then reached a plateau. In contrast, BM-MSC and UC-MSC transplantations caused improvements in mechanical allodynia (df: 8, 63; F = 1060.2; *p* < 0.001) so that in the seventh and eighth week, this threshold was not significantly different from that of the sham and control groups (*p* > 0.05) (Fig. 2b). There was no significant difference among stem cell treated groups (*p* >0.99)

#### Cold allodynia

The percentage of paw withdrawal responses of the animal to cold stimulation significantly increased after SCI induction, compared to the control and sham groups (df: 8, 63; F = 17.7; *p* < 0.001). In SCI and vehicle groups, the percentage of responses continued to increase. On the other hand, BM-MSC and UC-MSC transplantations caused improvements in the rats response to cold stimulation compared to the SCI group (df: 8, 63; F = 426.4; *p* < 0.001), although this threshold did not return to the normal level (*p* < 0.05) (Fig. 2c). There was no significant difference among stem cell treated groups (*p* >0.99)

#### Mechanical hyperalgesia

The Randall-Sellito test revealed that SCI caused a decrease in the animal pain threshold under painful mechanical stimulation (df: 8, 63; F = 16.5; *p* < 0.001) (Fig. 2d). Transplantation of BM-MSCs and UC-MSCs led to significant improvement of paw withdrawal threshold compared to the SCI group (df: 8, 63; F = 230.4; *p* < 0.001). There was no significant difference between transplanted animals (*p* > 0.99).

#### Heat hyperalgesia

As Fig. 2e shows, SCI led to a significant decrease in paw withdrawal threshold due to heat stimulation (df: 8, 63; F = 24.0; *p* < 0.001). BM-MSC and UC-MSC transplantations caused the threshold to rise from the second week onwards and reach the normal level in week 4 (df: 8, 63; F = 292.0; *p* < 0.0001). There was no significant difference among stem cell treated groups (*p* > 0.99).

### Histological evaluation

**Fig. 3:**
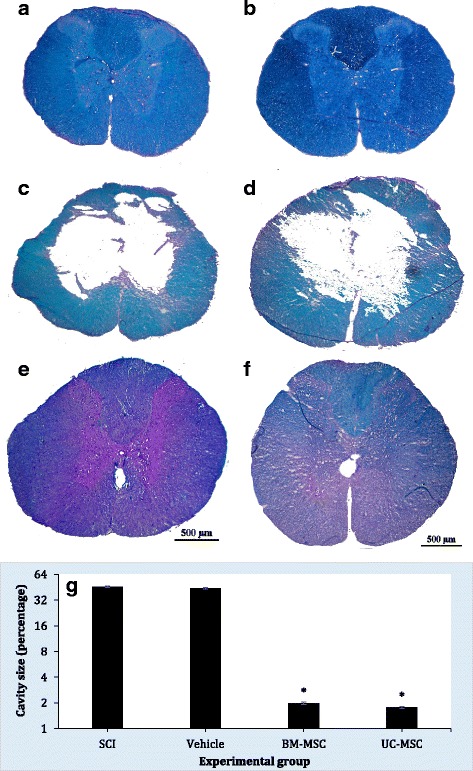
Luxol fast blue staining for assessment of cavity size in control (**a**), sham (**b**), spinal cord injured (*SCI*) (**c**), vehicle-treated (**d**), human bone marrow-derived mesenchymal stem cell (*BM-MSC*) (**e**) and umbilical cord-derived mesenchymal stem cell (*UC-MSC*) (**f**) animals. Transplantation of human BM-MSCs and UC-MSCs resulted in significantly decreased cavity size (**g**). Original magnification in **a**–**f**, ×20. Data are expressed as means ± SEM (n = 3 in each group). *p < 0.001, versus SCI group

A big cavity was seen in the SCI and vehicle groups 8 weeks post-SCI. However, BM-MSC and UC-MSC transplantations prevented cavity formation and SCI development (Fig. 3). The size of the cavity was significantly lower in BM-MSC and UC-MSC groups compared to SCI and vehicle groups (df: 16; F = 89.4; *p* < 0.001). However the size of the cavity was not different between transplanted animals (*p* > 0.99).

**Fig. 4:**
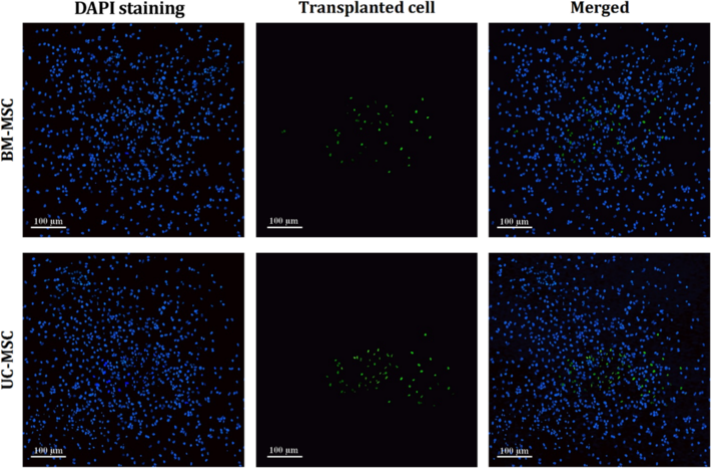
Immunohistochemistry staining for assessment of human bone marrow-derived mesenchymal stem cell (*BM-MSC*) and umbilical cord-derived mesenchymal stem cell (*UC-MSC*) survival. Host cells are stained by 4',6-diamidino-2-phenylindole (*DAPI*). Mouse monoclonal antibody against human nuclei positive cells (transplanted cells) continue to survive in the spinal cord after 8 weeks (n = 3 in each group). The survival rates were 0.36 ± 0.06 % and 0.57 ± 0.06 % in BM-MSC and UC-MSC groups, respectively (*p* = 0.002)

In addition, immunohistochemistry staining showed that transplanted cells continued to survive in the spinal cord after 8 weeks. In Fig. 4, transplanted cells can be seen in green. The survival rates were 0.36 ± 0.06 % and 0.57 ± 0.06 % in BM-MSC and UC-MSC groups, respectively (*p* = 0.01). The number of surviving cells in the BM-MSC and UC-MSC groups were 2327.0 ± 571.88 and 5728.67 ± 583.15, respectively (*p* = 0.002).

### Electrophysiological findings

For electrophysiological investigations, a single-unit recording of WDR neurons in the dorsal horn of the L4 and L5 spinal cord was obtained. The WDR neuron response to electrical stimulation was evaluated 8 weeks after transplantation of BM-MSCs and UC-MSCs. Compared to control and sham groups, evoked potentials of the WDR neurons in the SCI and vehicle groups were significantly higher. The response of these neurons to the stimulations received from Aβ (df: 35; F = 28.9; *p* < 0.001), Aδ (df: 35; F = 34.5; *p* < 0.001), and C (df: 35; F = 40.6; *p* < 0.001) fibers, as well as post-discharge response (df: 35; F = 31.5; *p* < 0.001) and wind up (df: 35; F = 30.6; *p* < 0.001) were higher in the SCI and vehicle groups compared to sham and control groups.

**Fig. 5:**
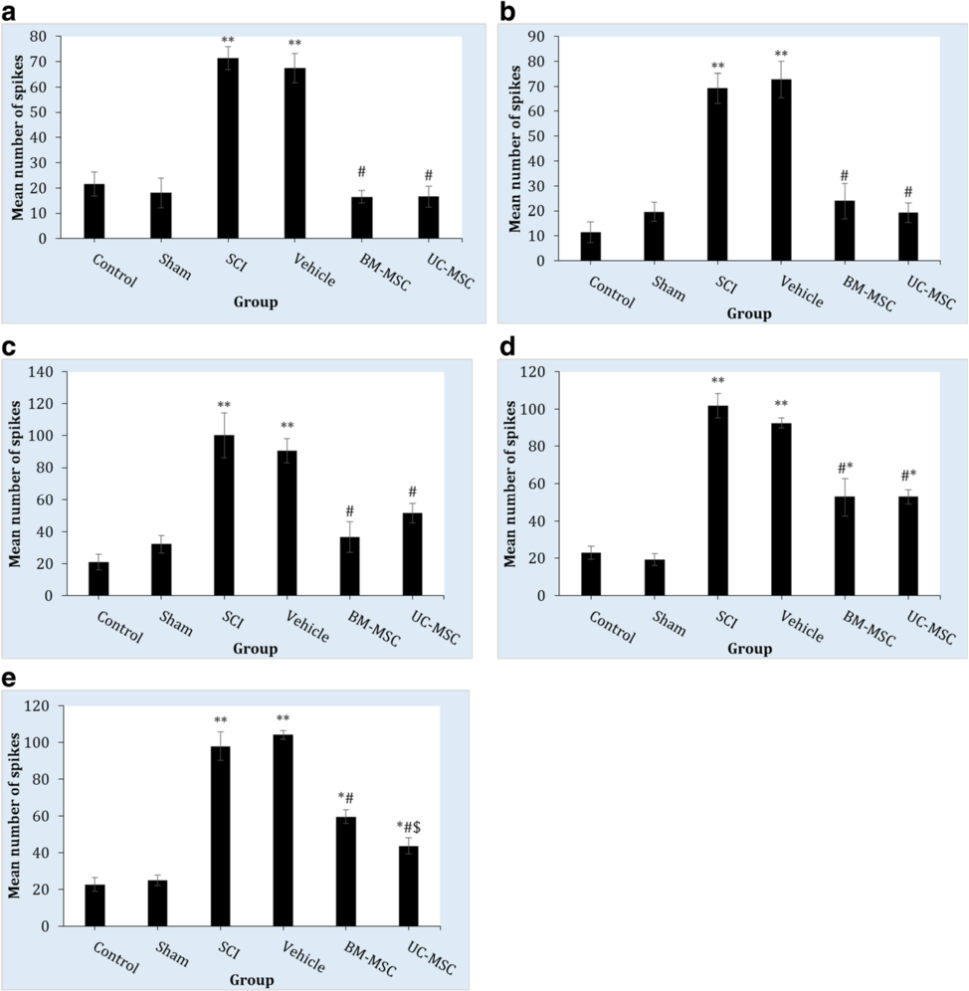
Single-unit recording of wide dynamic range (WDR) neurons in the dorsal horn of the L4 and L5 spinal cord 8 weeks after transplantation of human bone marrow-derived mesenchymal stem cells (*BM-MSCs*) and umbilical cord-derived mesenchymal stem cells (*UC-MSCs*). Evoked potential of the WDR neurons to stimulations received from Aβ neurons (**a**), Aδ (**b**), and C fibers (**c**), post-discharge response (**d**), and wind up (**e**) are presented as means ± SEM (n = 6 in each group). ^#^ *p* < 0.001, versus SCI group; *p < 0.01, **p < 0.001, versus sham animals; ^$^ *p* < 0.01, versus BM-MSC treated animals. *SCI* spinal cord injury

**Fig. 6:**
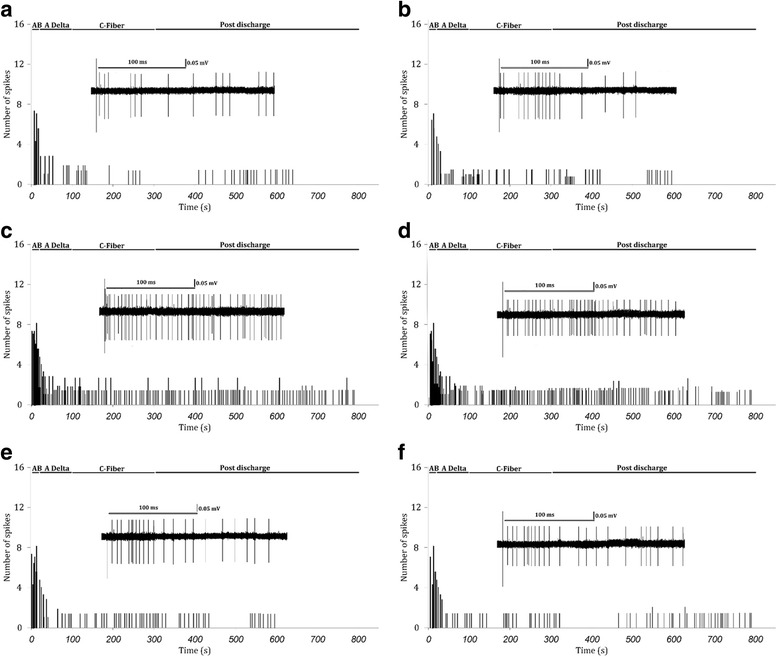
Examples of the raw evoked responses and post-stimulus time histogram of WDR neurons in control (**a**), sham (**b**), SCI (**c**), vehicle-treated (**d**), human BM-MSC (**e**) and UC-MSC (**f**) animals

BM-MSC treatment caused the WDR neuron response to stimulations from Aβ (*p* = 0.91), Aδ (*p* = 0.87) and C (*p* = 0.99) fibers to reach that of the control group. Although post-discharge and wind up became significantly lower than in the SCI group (*p* < 0.001), they did not reach the normal level (*p* < 0.05) (Figs. 5 and 6).

A similar pattern was observed regarding UC-MSC transplants. Transplantation of UC-MSCs led to WDR neuron response to stimulations from Aβ (*p* = 0.99), Aδ (*p* = 0.95) and C (*p* = 0.24) fibers reaching the control group level. However, UC-MSCs did not cause post-discharge and wind up to reach normal levels (*p* < 0.05) although they did significantly decrease compared to the SCI group (*p* < 0.001). Comparing the two treatments regimes revealed that wind up in the UC-MSC group was significantly lower than the BM-MSC group (*p* = 0.008).

## Discussion

The results of the present study showed that transplantation of BM-MSCs and UC-MSCs in the spinal cord alleviate the allodynia and hyperalgesia after SCI. The efficacy of the two types of cells was similar for symptom relief. These cells survive in the spinal cord and prevent formation of cavities due to SCI. However, the survival rate of UC-MSCs was significantly higher than BM-MSCs. Electrophysiological evaluation confirmed these findings. Evoked response of the WDR to Aβ, Aδ, and C fiber stimulations, post-discharge and wind up of these second order neurons (WDR) had significantly increased 8 weeks after SCI, while stem cell transplantation decreased the responses to painful stimulation. Statistical analysis showed that animals transplanted with UC-MSCs had better recovery in wind up phenomena. Although the pain threshold in animals transplanted with UC-MSCs and BM-MSCs was not at the level of normal animals, this might be due to an inability of these cells to fully recover post-discharge and wind up phenomena when transplanted into the spinal cord.

Efficacy of MSC transplantation on neuropathic pain depends on numerous factors, such as source of the cells (donor species) [44], number and source of transplanted cells, route of administration (at injury site or intravascular), type of injury (central or peripheral), type of transplant (allogenic or xenogenic), time between injury and cell transplantation, and follow-up duration [45–48]. This explains why MSC transplantation has led to neuropathic pain symptom relief in some studies [44, 45, 47, 49–58] but not others [48, 58–60], or even symptom worsening [46, 48]. The results of the present study revealed that transplantation of 1 million mesenchymal cells derived from bone marrow, umbilical cord, and adipose tissue led to neuropathic pain symptom relief after transplantation. To our knowledge, the present study is the first in vivo study comparing the efficacy of MSCs derived from different sources on motor recovery and neuropathic pain symptom relief following SCI. Therefore, our results cannot be directly compared to other studies. However, Jin et al. [61] compared the ability of mesenchymal cells derived from bone marrow, umbilical cord, and adipose tissue to differentiate to various tissues in an in-vitro study. They found that mesenchymal cells derived from umbilical cord had stronger proliferation ability and anti-inflammatory effects compared to other cells. They suggest that cells derived from umbilical cord have an advantage over those derived from adult tissues (such as bone marrow and adipose tissue) and can be used as an efficient model in the clinic [61]. In addition, Kern et al. [62] showed that, from morphologic and immune phenotype points of view, BM-MSCs and UC-MSCs are not significantly different. Although the number of colonies was lower in cells isolated from umbilical cord, in the next passages their proliferation and survival was higher than other studied cells [62]. Baksh et al. [63] compared proliferation and differentiation of mesenchymal cells isolated from bone marrow and umbilical cord and revealed that umbilical cord mesenchymal cells keep their mesenchymal characteristics for longer and express signaling pathways similar to those of mesenchymal cells isolated from bone marrow. Panepucci et al. [64] compared gene expression characteristics of mesenchymal cells isolated from bone marrow and umbilical cord and demonstrated that bone marrow-derived cells tend to express genes related to antimicrobial and osteogenesis processes, while umbilical cord-derived cells tend to express genes playing a part in angiogenesis and intracellular matrix renewal. Therefore, it can be suggested that, since mesenchymal cells isolated from umbilical cord have a higher survival rate in the tissue and show more anti-inflammatory and angiogenic effects compared to stem cells derived from bone marrow, they are expected to have higher efficacy in injury healing and symptom relief in neuropathic animals. This might be the reason that wind up recovery in the group treated with UC-MSCs was better than in the group treated with BM-MSCs in this study.

The present study showed that BM-MSCs and UC-MSCs are able to survive until 8 weeks after transplantation. Mannoji et al. [65] also reported similar results in this regard. Kim et al. [66] evaluated the effect of bone marrow-derived stem cell transplantation on SCI in rats and determined that these cells are present at the site of injury until 6 weeks after transplantation. They stated that the expression level of neuronal growth factor (NGF) and brain-derived neurotrophic factor (BDNF) in the group treated with mesenchymal cells was higher than their SCI group [66]. In addition, Veeravalli et al. [67] evaluated the effect of umbilical cord-derived stem cell transplantation and revealed that these cells were present at the site of injury at the 3-week follow-up. Transplantation of these cells had induced metalloproteinase 2 expression at the site of injury. Inhibition of this cellular matrix protein resulted in a decrease in the protective effect of mesenchymal cells. They concluded that transplantation of umbilical cord-derived stem cells prepares the environment for endogenic regeneration by inducing metalloproteinase 2 expression and inhibition of glial scar formation [67].

These studies all indicated that MSCs are able to survive in the tissue for a long time. Most studies show that these cells have a protective role in the tissue and may reduce inflammation caused by SCI. By secretion of cytokines and growth factors, MSCs can also play a role in neural regeneration [68]. Thus, it seems that they provide a favorable environment for endogenic regeneration. In the present study, LFB staining showed that MSC transplantation from two studied sources prevented cavity formation at the site of injury, which might be due to their anti-inflammatory role.

Two inflammation phases are present in SCI. A primary or acute phase at the time of injury causes some cells to die or experience ischemia due to direct compression or a decrease in blood flow. However, the major damage is done in the second phase. This phase lasts weeks or months, and causes spinal cord tissue damage by various mechanisms: apoptosis induction, initiation of astroglial scar formation, central chromatolysis, deficiency in myelin gene expression, degradation of myelin in remaining axons, glutamate over-induction, invasion of immune cells to the site of injury and secretion of inflammatory cytokines, and endothelial damage due to ischemia–reperfusion, and so forth. [69]. Mesenchymal cell transplantation can reverse these damaging processes to some extent. These cells have immunomodulatory characteristics [21, 70–75] and can minimize inflammation and immune system-induced damage if transplanted at the right time [76]. Transplantation of these cells can decrease glial cell hypertrophy and proliferation, and improve recovery with the aid of bioactive molecules, reduction of cytokine secretion, and growth factors. Their angiogenic role can also be used in angiogenesis in the spinal cord [77, 78].

To our knowledge, this study is the first to compare the electrophysiological changes of the spinal cord after BM-MSC and UC-MSC transplantation. The findings of the present study showed that SCI leads to an increase in stimulations from Aβ, Aδ, and C fibers to WDR neurons at below-injury levels, and that MSC treatment recovered these changes to the level of those of intact groups. It has been reported that transplantation of olfactory cells resulted in improvement in touch stimulations and cord dorsum potentials [79]. It was also shown that SCI at the T3 level resulted in complete neural disconnection between the parts on either side of the SCI site. However, after neural stem cell transplantation, an electrical stimulation at the C7 level led to recording of an evoked potential in the T6 area [80]. Erceg et al. [81] showed that transplantation of oligodendrocyte and motoneuron progenitor cells into injured spinal cord could result in restoration of motor pathways that were damaged due to SCI to some extent, and lead to appearance of motor-evoked potential in electrophysiological recordings [81]. Yasuda et al. [82] also revealed that neural stem/progenitor cell transplantation brings about reappearance of motor-evoked potential post-SCI. In addition, Ziegler et al. [83] reported that olfactory ensheathing glial cell transplantation after complete SCI led to reappearance of motor-evoked potentials. In another study, comparison of electrophysiological findings in a rat model showed that somatosensory evoked potential in the BM-MSC treated group was not different from the Schwann cell group [84].

Finally, we should note that, in this study, about 1 million cells were transplanted a week after injury. The reason for the selection of this protocol was its similarity to clinical conditions. In the clinical trials performed recently, mean injected cell count was about 1–3 million cells [85–88]. Additionally, in most clinical conditions, transplantation of cells at the time of injury is not possible and preparation of the patients and cells for transplantation takes at least 1 week to a few months. We cannot determine the mechanism(s) that led to the improvement observed. The cells might have prevented inflammation and stopped the damaging process in the initial days with no further function after that or, on the contrary, they could have continued to exert a protective role. Further study is needed to determine this.

The present study has some limitation. First, the cells are cultured in fetal bovine serum, which is not ideal for application in humans. However, using fetal bovine serum is in accordance with cell culture protocols. Therefore, when translating the result to a clinical trial an alternative for fetal bovine serum will be needed. Second, we compared the efficacy of BM-MSC and UC-MSC xenotransplantation on improvement of SCI 1 week after induction of compression injury. Since we showed the efficacy of BM-MSCs are affected by timing of intervention, the method used for SCI induction, and dosage of cell therapy in a previous meta-analysis [14], generalizing our findings should be done with caution.

## Conclusion

The results of the present study showed that BM-MSC and UC-MSC transplantations alleviated the symptoms of neuropathic pain and resulted in subsequent motor recovery after SCI. Efficacy of both sources was similar for symptom relief. These cells survived in the tissue at least 8 weeks and prevented cavity formation due to SCI. However, the survival rate of UC-MSCs was significantly higher than BM-MSCs. Electrophysiological evaluations showed that transplantation of UC-MSCs brings about better results than BM-MSCs in wind up of WDR neurons.

## Abbreviations

BBB: Basso, Beattie, and Bresnahan
BM-MSC: Bone marrow-derived mesenchymal stem cell
DMEM: Dulbecco's modified Eagle's medium
LFB: Luxol fast blue
MSC: Mesenchymal stem cell
PBS: Phosphate-buffered saline
SCI: Spinal cord injury
UC-MSC: Umbilical cord-derived mesenchymal stem cell
WDR: Wide dynamic range
